# Oxalate nephropathy after pancreaticoduodenectomy: a case report

**DOI:** 10.1186/s12882-024-03543-9

**Published:** 2024-03-18

**Authors:** Claire Barani, Selda Aydin, Nathalie Demoulin, Michel Jadoul

**Affiliations:** 1 Division of Nephrology, Cliniques Universitaires Saint-Luc, Université Catholique de Louvain, Avenue Hippocrate 10, B-1200 Brussels, Belgium; 2 Division of Pathology, Cliniques Universitaires Saint-Luc, Université Catholique de Louvain, Avenue Hippocrate 10, B-1200 Brussels, Belgium

**Keywords:** Case report, Oxalate nephropathy, Oxaluria, Pancreatectomy

## Abstract

A 75-year-old male developed acute kidney injury KDIGO stage 3 a few weeks after Whipple surgery was performed for a distal cholangiocarcinoma. Kidney biopsy revealed oxalate nephropathy. This was attributed to post-Whipple malabsorption, poor compliance with pancreatic enzyme replacement therapy, and daily intake of vitamin C supplements. Pancreatic enzyme replacement therapy was resumed and calcium carbonate initiated, with an improvement in glomerular filtration rate. Unfortunately, due to oncological progression, best supportive care was initiated.

We review the pathophysiology and conditions predisposing to secondary hyperoxaluria and oxalate nephropathy. This diagnosis should be considered among the main causes of acute kidney injury following pancreatectomy, with important therapeutic implications.

## Case report

We report the case of a 75 -year-old male patient with acute kidney injury (AKI) KDIGO stage 3 shortly after pancreatectomy. His medical history included essential hypertension treated by perindopril/indapamide, and benign prostatic hyperplasia.

In Augustus 2021, he presented with jaundice and abdominal pain. A diagnosis of distal cholangiocarcinoma was made. He received antibiotic therapy for suspicion of sepsis and a non-covered metal biliary stent was placed. He presented during this hospital stay an acute oliguric kidney injury (peak creatinine: 8.59 mg/dl) ascribed to acute tubular necrosis associated with biliary sepsis and bilirubin toxicity. At discharge, serum creatinine was 2.83 mg/dl, decreasing gradually to 1.36 mg/dl in January. He underwent Whipple surgery on January 18th, 2022. Plasma creatinine at discharge on postoperative day 7 was 1.35 mg/dl (baseline 1.1 mg/dl)and remained stable throughout the month of February. Blood glucose remained consistently normal.

In March 2022, acute kidney injury was detected on the day of a planned adjuvant chemotherapy with capecitabin. Laboratory tests showed: creatinine 5.43 mg/dl, glucose 85 mg/dl, potassium 5.6 mmol/l, bicarbonate 19 mmol/l, calcium 2.36 mmol/l, phosphate 1.87 mmol/l, AST 91 U/l, ALT 202 U/l, Hb10 g/dl, platelets 118.000/µl. Urine testing showed an albumin creatinine ratio of 21.5 mg/g (*N* < 30). There was neither hematuria nor leucocyturia nor urinary crystals but urinary oxalate level was increased at 61 mg/g creatinine (Nl < 50)). Fractional excretion of sodium was 2.2%, suggesting a parenchymatous kidney disease. The patient had no complains except diarrhea alternating with constipation. His physical examination was normal except a mild lower limb oedema. His medications included pancreatin 420 mg three times per day, cholecalciferol 25.000 U once a week, pantoprazole 20 mg once a day, an over-the counter supplement of Vitamin C (unknown exact dosage) and Perindopril/Indapamide 2.5 mg/0.625 mg once a day. Both vitamin C and perindopril/indapamide were discontinued. An ultrasound of the kidneys showed no sign of obstruction of the urinary tract, nor signs suggestive of renal artery stenosis.

**Fig. 1:**
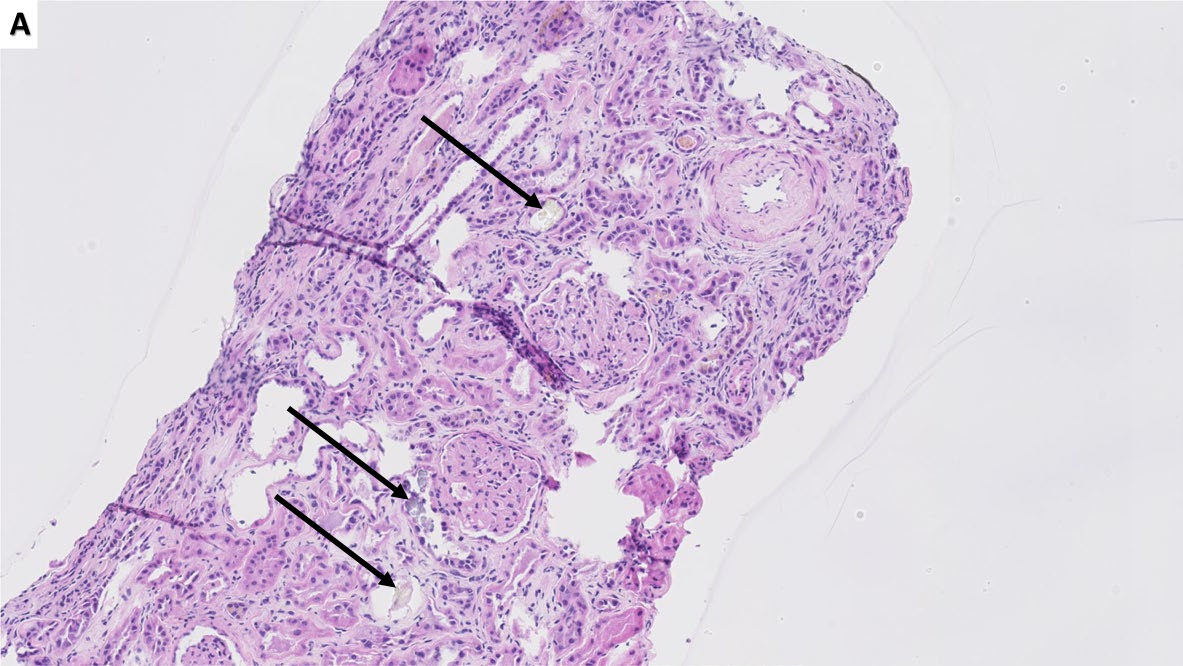
Kidney biopsy sample showing several intratubular calcium oxalate crystals (black arrows) under light microscopy (hematoxylin and eosin stain, original magnification × 10)

**Fig. 2:**
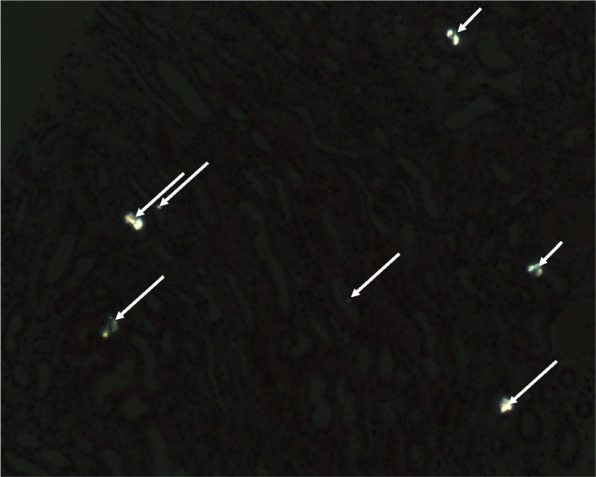
Multiple intratubular translucent crystals showed birefringence under polarized light and a radial pattern distribution

A kidney biopsy confirmed our suspicion of oxalate nephropathy: eleven intratubular translucent crystals were detected (Fig. 1). They showed birefringence under polarized light and a radial pattern distribution (Fig. 2). Some foci of acute interstitial nephritis and acute tubular necrosis were present. Oxalate nephropathy was attributed to hyperoxaluria of multiple origin: post-Whipple malabsorption favored by poor compliance with pancreatic enzyme replacement therapy, combined with daily intake of a supplement of Vitamin C. Treatment consisted of high fluid intake and oral calcium carbonate supplements. The patient was advised to take the pancreatic enzymes with every meal. Serum creatinine decreased to 3.28 mg/dl. Unfortunately, peritoneal carcinomatosis developed with secondary cholangitis and best supportive care was initiated.

## Discussion

**Fig. 3:**
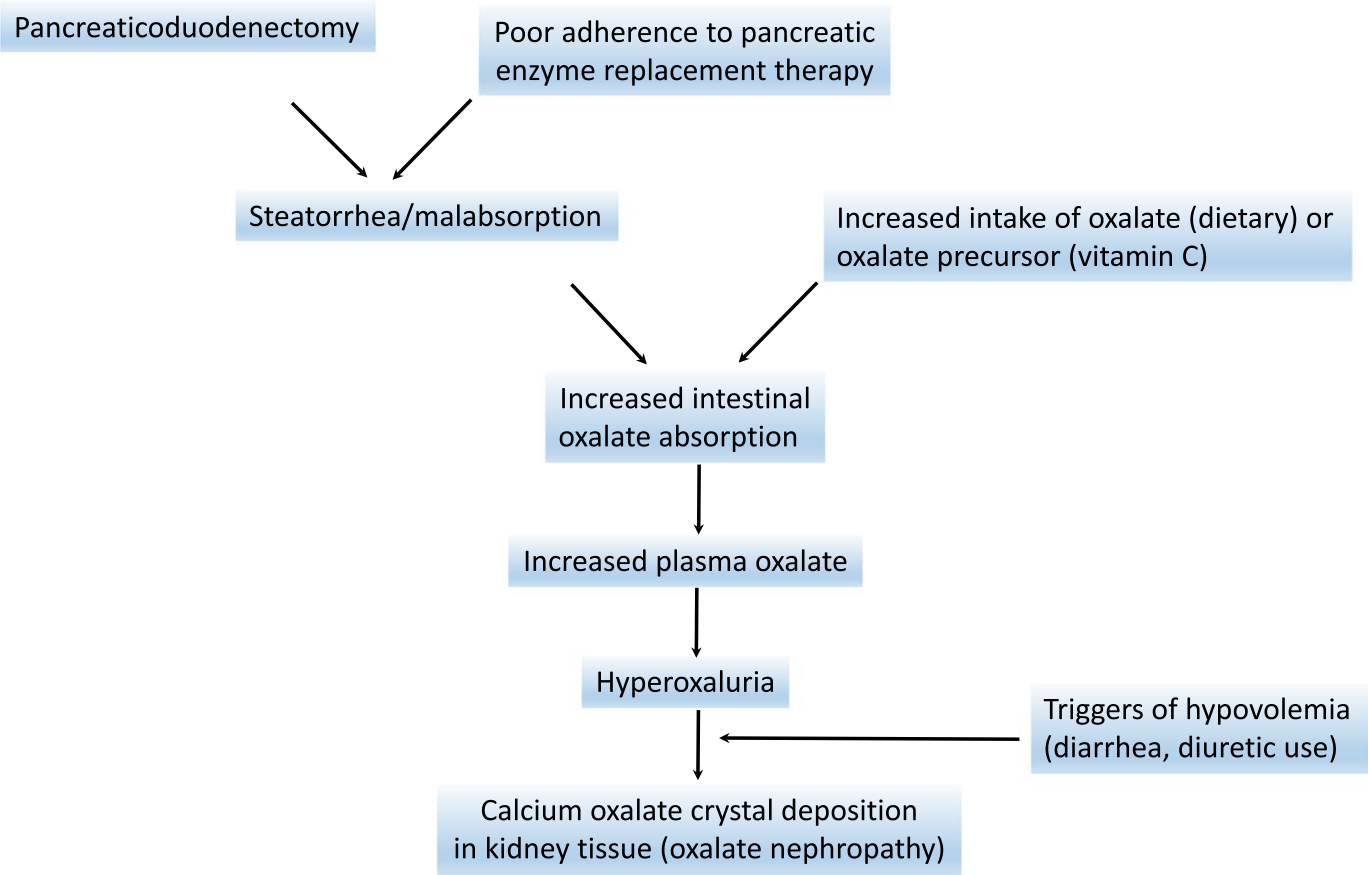
Pathophysiology of hyperoxaluria and oxalate deposition in the kidney in the reported case

Hyperoxaluria results from inherited disorders of glyoxylate metabolism (primary hyperoxaluria) or increased intestinal oxalate absorption (secondary hyperoxaluria) [1]. Primary hyperoxaluria types 1, 2 and 3 are caused by pathogenic variants in *AGXT*, *GRHPR* or *HOGA1*. Secondary hyperoxaluria can be due either to increased intake of oxalate or oxalate precursor(s), or fat malabsorption, or decreased intestinal oxalate degradation. It often results from a combination of these factors, and acute dehydration, diuretic use, inflammation and antibiotic use may further increase the urinary oxalate concentration [1]. In our case, although the intermittent diarrhea and diuretic treatment (both potentially causing hypovolemia), as well as pantoprazole treatement may have played some role in the lesions of acute tubular necrosis and interstitial nephritis, the kidney biopsy pointed to the key cause of acute kidney injury, by showing numerous oxalate crystals in the kidney, despite the absence of oxalate crystals at urinalysis. The underlying pathophysiology is clear: there was no history of gastric bypass, orlistat use of small bowel disease (Crohn’s disease of post-surgical short bowel), but pancreatectomy caused an abrupt exocrine pancreatic insufficiency, leading to fat malabsorption (Fig. 3). As a result, free fatty acids bind in the bowel lumen to calcium. There is thus less calcium available to bind oxalate in the bowel, resulting in increased absorption of free oxalate. In our patient hyperoxaluria also resulted from daily intake of ascorbic acid, a precursor of oxalate: circulating ascorbic acid is converted to dehydroascorbate then to diketogluconic acid, that breaks down to oxalate [2]. Hyperoxaluria may then lead to urinary supersaturation of calcium oxalate and crystal formation, contributing to nephrolithiasis and deposition of oxalate crystals in renal tissue (oxalate nephropathy). The latter causes tubulo-interstitial injury and fibrosis, acute kidney injury and/or chronic kidney disease [1]. It may be worth mentioning here that the sensitivity of urinary oxalate level may be decreased in case of kidney failure, precisely because of the lower urinary elimination of oxalate, as a result of tissue deposition.

Oxalate nephropathy caused by exocrine pancreatic insufficiency is well-documented [3, 4]; the review of 2265 native kidney biopsies identified ON as the cause of kidney injury in 1% of cases, with chronic pancreatitis being the cause in 24% of them [4].

Oxalate nephropathy following pancreatic surgery has received hitherto little attention. A single retrospective study of pancreatectomies for adenocarcinoma performed between 2010 and 2017, identified oxalate nephropathy in 1% of patients but this was based on 3 cases only [3]. The risk was higher after pancreaticoduodenectomy than after splenopancreatectomy.

Cases of vitamin C induced oxalate nephropathy have been reported after oral and IV use of vitamin C in the intensive care unit as an adjunctive therapy for sepsis [5]. Interestingly, the COVID-19 pandemic prompted greater use of vitamin C supplements and was accompanied by an increase of cases of oxalate nephropathy secondary to high-dose vitamin [6]. The dose and duration of vitamin C therapy associated with oxalate nephropathy is variable.

Also, a multifactorial origin of ON, including an association with both vitamin C intake and malabsorption has already been described [7–9] but to the best of our knowledge, this is the first case reporting a combination of pancreatectomy and intake of vitamin C leading to acute oxalate nephropathy.

The prognosis of oxalate nephropathy associated with secondary hyperoxaluria is variable, with about 50% of patients reaching kidney failure [1, 4]. Treatment includes high fluid intake, calcium supplements to limit the bioavailability of intestinal oxalate, and management of the underlying cause of hyperoxaluria.

In conclusion, oxalate nephropathy should be considered among the causes of acute kidney injury following pancreatectomy, with important therapeutic implications.

## Data Availability

The availability of anonymized data presented in this case report can be obtained from the corresponding author (MJ) upon motivated request.
